# Calidad de la espirometría forzada a 2.700 msnm en adultos sanos

**DOI:** 10.7705/biomedica.7804

**Published:** 2025-09-22

**Authors:** Adriana Sofía Valero-Ortiz, Myriam Rocío Wilches-Wilches, Marcela América Roa-Cubaque, Clara Lizeth Palencia-Mojica, Flor Ángela Umbacía-Salas, Alba Yanira Polanía-Robayo, Mónica Paola Quemba-Mesa, Paola Delgado-Villalba, Ángela Marcela Flechas-Robles

**Affiliations:** 1 Facultad Ciencias de la Salud, Universidad de Boyacá, Tunja, Colombia Universidad de Boyacá Facultad Ciencias de la Salud Universidad de Boyacá Tunja Colombia; 2 Laboratorio de Pruebas, Fundación Neumológica Colombiana, Bogotá, D. C., Colombia Laboratorio de Pruebas Fundación Neumológica Colombiana Bogotá D. C Colombia

**Keywords:** espirometría, altitud, pruebas de función respiratoria, mecánica respiratoria, capacitación profesional, técnicas de diagnóstico del sistema respiratorio, spirometry, altitude, respiratory function tests, respiratory mechanics, professional training, diagnostic techniques, respiratory system

## Abstract

**Introducción.:**

La espirometría forzada es una prueba para evaluar la mecánica respiratoria según los criterios de la *American Thoracic Society* y la *European Respiratory Society* en su actualización del 2019. La calidad de la prueba depende de la aceptabilidad y repetibilidad de las curvas obtenidas, además de factores ambientales, como la altura sobre el nivel del mar.

**Objetivo.:**

Determinar la calidad de las pruebas de espirometría forzada en adultos sanos practicadas a 2.700 msnm.

**Materiales y métodos.:**

Se llevó a cabo un estudio prospectivo y cuantitativo de cohorte sobre los valores de referencia de la espirometría forzada a 2.700 msnm de una población sana. Se evaluó la calidad de las pruebas mediante los criterios de aceptabilidad y repetibilidad.

**Resultados.:**

Se evaluaron 417 espirometrías forzadas en adultos sanos, practicadas a 2.700 msnm. El 81 % de las pruebas cumplió los criterios de calidad. Se encontró mayor incumplimiento en los criterios de aceptabilidad, específicamente, en el "buen inicio" de la maniobra, en el cual el tiempo para alcanzar el pico de flujo espiratorio fue mayor de 150 ms, seguido por la presencia de artefactos, principalmente el cierre glótico, y en el de "buen final" debido a la terminación temprana de la espiración.

**Conclusiones.:**

A medida que avanzaron las sesiones, se optimizó la calidad de las espirometrías forzadas, favorecida por el perfeccionamiento de la técnica y la capacitación continua de los profesionales.

## Abstract (en)

**Introduction.:**

Forced spirometry evaluates respiratory mechanics according to the 2019 American Thoracic Society and European Respiratory Society criteria. The quality of the test depends on the evaluation of the curves obtained according to acceptability and repeatability, in addition to environmental factors such as altitude above sea level.

**Objective.:**

To determine the quality of forced spirometry tests in a healthy adult population at 2,700 meters above sea level.

**Materials and methods.:**

We conducted a prospective quantitative cohort study on reference values for forced spirometry in a healthy population at 2,700 meters above sea level. Test quality was evaluated using acceptability and repeatability criteria. Based on a prospective quantitative cohort study of forced spirometry reference values in a healthy respiratory population at 2,700 meters above sea level, the quality of the tests was evaluated considering the acceptability and repeatability criteria.

**Results.:**

We performed 417 forced spirometry tests in a healthy adult population at 2,700 meters above sea level, 81% of which fulfilled the quality criteria. A greater proportion of tests did not meet the acceptability criteria, specifically, "the good start" item due to a time higher than 150 ms to achieve the peak expiratory flow; followed by artifacts such as glottic closure, the most frequent; and "the good end" criterion due to an early termination of exhalation.

**Conclusions.:**

As the sessions progressed, the quality of the forced spirometry tests was optimized, a condition favored by the improvement of the technique and continuous training of the professionals.

La espirometría forzada es una prueba diagnóstica de la función pulmonar que permite evaluar la mecánica respiratoria ^1^. La calidad de la prueba depende de las especificaciones técnicas de los equipos, las variables de corrección y los criterios de aceptabilidad y repetibilidad ^2^. De igual manera, la validez de las pruebas requiere de personal técnico entrenado, que garantice un examen apropiado según los criterios de aceptabilidad y repetibilidad consignados en la actualización del 2019 de la guía de la *American Thoracic Society* (ATS) y la *European Respiratory Society* (ERS) ^3^.

Los principales errores que determinan el incumplimiento de los criterios de aceptabilidad y repetibilidad de una prueba espirométrica, surgen de la inexperiencia laboral de los técnicos, razón por la cual requieren capacitaciones estandarizadas para minimizar tales errores. Las alteraciones más comunes detectadas por el técnico son: aumento del tiempo para alcanzar el pico de flujo espiratorio, volumen retroextrapolado, capacidad vital inspiratoria forzada, tiempo espiratorio forzado, fin de la espiración forzada, tos en el primer segundo durante la medición del volumen espiratorio forzado, cierre glótico, introducción de la lengua en la boquilla y exhalación con una duración inferior a quince segundos, entre otros. También, se identificaron errores relacionados con la repetibilidad de las pruebas, lo que genera inconsistencias en los resultados de las espirometrías ^4^.

Por otro lado, se requiere un mínimo de tres maniobras por sesión y hasta un máximo de ocho para obtener una prueba adecuada ^5^. Sin embargo, existe la posibilidad de deterioro progresivo del rendimiento en cada sesión debido a la acumulación de fatiga muscular respiratoria, lo que influye en la toma inexacta de las mediciones y en la interpretación errónea de los criterios de calidad ^1^.

El registro adecuado de variables como la altitud, la humedad relativa y la presión barométrica, es importante en el ajuste previo de los equipos, ya que influyen directamente en la determinación de los volúmenes y capacidades dinámicas, como la capacidad vital forzada (CVF), el volumen espiratorio forzado en el primer segundo (VEF_1_) y en la relación entre estos dos parámetros ^6^^,^^7^. Por otra parte se ha demostrado que la medición de la CVF se ve afectada en las poblaciones que residen en grandes altitudes, además de disminuir sus valores cuando las tomas se realizaban durante la mañana y sin evidenciar mejoría tras la aclimatación ^8^.

La espirometría es una herramienta útil, tanto en la atención primaria como en la especializada, que abarca desde el diagnóstico y el seguimiento de enfermedades respiratorias, hasta el tamizaje y la evaluación de intervenciones terapéuticas. Por esta razón, es fundamental que su ejecución e interpretación sean adecuadas, de forma que permita valoraciones objetivas y decisiones terapéuticas acertadas ^9^.

En concordancia con lo expuesto, el objetivo de este estudio fue determinar la calidad de las pruebas de espirometría forzada a 2.700 msnm en adultos sanos. Esta investigación se enmarca en el proyecto denominado: "Valores de referencia en espirometría forzada en población adulta sana residente en Tunja, Colombia".

## Materiales y métodos

Se desarrolló un estudio observacional con enfoque cuantitativo y de corte transversal analítico, cuyo objetivo fue determinar la calidad de las pruebas de espirometría forzada a 2.700 msnm en adultos sanos. Las pruebas se realizaron, según las guías ATS/ERS del 2019, y fueron practicadas por profesionales en terapia respiratoria, entrenados en la ejecución e interpretación de la espirometría. La calidad de las pruebas fue evaluada por expertos en función pulmonar de la Fundación Neumológica Colombiana.

Los equipos utilizados fueron dos espirómetros portátiles ultrasónicos marca EasyOne™ (Medical Technologies, Zurich, Suiza), cuya calibración se verificó diariamente con una jeringa de 3 litros de acuerdo con las especificaciones técnicas de la norma de calidad ISO 26782.

Se excluyeron del estudio personas con índice de masa corporal (IMC) mayor de 30 kg/m^2^, fumadoras o con antecedentes de tabaquismo, embarazadas o con limitación cognitiva; en cuanto a las pruebas, no se incluyeron las espirometrías que no cumplían los criterios de aceptabilidad, repetibilidad y calidad, según la ATS.

### Población de estudio

La población estudiada incluyó adultos entre los 18 y los 80 años, residentes en Tunja, a quienes se les aplicó el instrumento usado para el estudio Proyecto Latinoamericano de Investigación en Obstrucción Pulmonar (PLATINO), útil para la identificación de personas sin alteraciones respiratorias a partir de la indagación de antecedentes y síntomas de enfermedad respiratoria. Se hizo un muestreo no probabilístico por conveniencia, reclutando participantes que cumplían los criterios de inclusión.

### Consideraciones éticas

El protocolo fue aprobado por el Comité de Ética de la Fundación Neumológica Colombiana, y el Comité de Ética y Bioética de la Universidad de Boyacá. Cada participante firmó un consentimiento informado, en el cual se garantizaba la confidencialidad de los datos y el uso exclusivo de la información con fines investigativos.

## Resultados

Se practicaron 417 pruebas, de las cuales 339 cumplían con los criterios de calidad, mientras que 78 fueron excluidas. Respecto a las características demográficas de la población estudiada, la edad promedio fue de 40 años (DE = 15,2), con una máxima de 88 y una mínima de 18 años; el sexo predominante fue el femenino (cuadro 1).

**Cuadro 1:** Características demográficas de la población estudiada

Variable		N	x̅	DE	IC_95%_	
					Min.	Max.
Edad		417	40	15,2	38	41
Sexo						
	Masculino	169	40,5		36	45,1
	Femenino	247	59,2		54,4	63,8

x̅: promedio; DE: desviación estándar; IC 95 %: intervalo de confianza del 95 %

### Pruebas que cumplieron con los criterios de calidad

**Figura 1:**
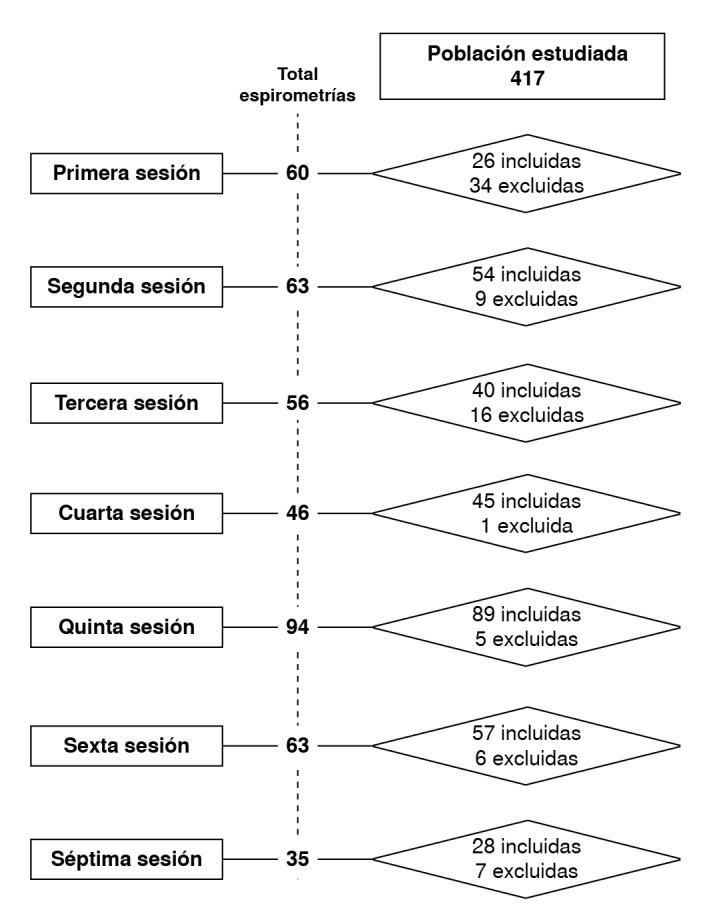
Flujograma de espirometrías forzadas

En la figura 1 se muestra la secuencia de ejecución de las pruebas, organizada en siete sesiones. En cada sesión se especificaron las pruebas incluidas y las excluidas. Se puede inferir que los terapeutas respiratorios mejoraron en la práctica de las pruebas a partir de las asesorías técnicas y la identificación de los errores más comunes, lo que permitió lograr espirometrías de calidad.

### Criterios de aceptabilidad y repetibilidad de las pruebas

Durante el análisis de las pruebas se observó que algunas presentaban uno o varios errores (cuadro 2).

**Cuadro 2:** Criterios de aceptabilidad y repetibilidad de las espirometrías

Criterio no cumplido		n	(%)
Criterios de aceptabilidad		44	(100)
	Buen Inicio		
	Pico de flujo espiratorio pico > 150 ms	39	(88,6)
	Volumen extrapolado	4	(9,09)
	CVI > CVF	1	(2,27)
Ausencia de artefactos		42	(100)
	Cierre de glotis	36	(85,7)
	Esfuerzo variable	6	(14,2)
Buen final		32	(100)
	Terminación temprana	28	(87,5)
	Fugas	4	(12,5)
Criterios de repetibilidad		14	(100)

CVI: capacidad vital inspiratória; CVF: capacidad vital forzada

*Aceptabilidad de la prueba:* la aceptabilidad fue evaluada mediante los siguientes criterios:

- Buen inicio basal: se refiere a un inicio adecuado de la maniobra de espirometría, visualizado en la curva flujo como un inicio rápido, vertical, sin vacilaciones, un pico de flujo espiratorio en un tiempo menor de 150 ms y un volumen retroextrapolado retrógrado menor de 150 ml o al 5 % de la capacidad vital forzada ^10^. Según este criterio, se identificaron 44 pruebas con errores. El error más frecuente fue alcanzar el pico de flujo espiratorio en un tiempo mayor de 150 ms, seguido de un volumen retroextrapolado mayor del 5 % de la capacidad vital forzada y, en menor proporción, de una capacidad vital inspiratoria superior a la capacidad vital forzada.
- Ausencia de artefactos: se refiere a situaciones en las que el individuo no realiza de manera adecuada la maniobra, como terminación temprana de la prueba, esfuerzos submáximos, tos en el primer segundo de la espiración, dobles inspiraciones o espiraciones, obstrucción de la boquilla con la lengua, cierre glótico y fugas de aire (pérdida de volumen) ^11^. A causa de este criterio, se excluyeron 42 pruebas por presencia de artefactos; los más frecuentes fueron el cierre de la glotis y un esfuerzo espiratorio variable.
- Buen final: se refiere a la finalización del esfuerzo espirtatorio, el cual se evalúa según tres condiciones: cambio del volumen pulmonar inferior a 25 ml durante, al menos, 1 segundo (corresponde a la meseta visible en la curva); una espiración forzada que alcance el límite de 15 s ^3^, y la ausencia de terminación temprana o fugas de aire. Por este criterio, se excluyeron 32 pruebas con terminación temprana y fugas de aire.

*Repetibilidad de la prueba:* es la concordancia entre las mediciones de capacidad vital forzada y volumen espiratorio forzado en el primer segundo. Su valor debe ser inferior a 150 ml entre las dos mejores pruebas. Se identificaron 14 pruebas que no cumplieron con el criterio de repetibilidad.

## Discusión

En el presente estudio se evidenció que, de las pruebas de espirometría forzada que pasaron los criterios de calidad, el 81 % cumplió con los criterios de aceptabilidad y repetibilidad establecidos por las guías ATS/ERS del 2019, lo que indica una adecuada precisión en la ejecución de las pruebas. Sin embargo, el 19 % restante se excluyó principalmente por no tener un buen inicio de la maniobra -con un tiempo de alcance del pico de flujo espiratorio mayor de 150 ms- y por la presencia de artefactos, entre los que el cierre glótico fue el más frecuente. También, se observó terminación temprana de la espiración en algunas pruebas, lo que comprometió su validez. En términos de repetibilidad, 14 pruebas no cumplieron con este criterio.

Los resultados de este estudio destacan la importancia de la estandarización de la espirometría forzada y la capacitación continua de los técnicos. Rodríguez Rocha *et al.,* en un estudio en Tenerife, reportaron que el 45,7 % de las espirometrías fueron inaceptables, principalmente por los artefactos observados en la curva espiratoria ^12^. De manera similar, Alavi Foumani *et al.* encontraron que el 69,7 % de las pruebas realizadas en el ámbito de la salud ocupacional fueron inválidas debido a errores técnicos relacionados con la repetibilidad y la ejecución de la maniobra ^13^. Al comparar los resultados de estos estudios con los del presente, se evidencia una problemática común en cuanto a la precisión técnica de las pruebas de espirometría.

En la investigación realizada por San Martín, en Asunción, el 61,4 % de los evaluados correspondió al sexo femenino y la edad de la población estudiada osciló entre 18 y 77 años. Estos resultados de distribución por sexo y edad, son similares a los del presente estudio. De igual manera, la terminación temprana de la espirometría fue el criterio de calidad con mayor error en los dos estudios ^14^.

En el estudio de Enright *et al.*^15^, sobre la calidad de las pruebas de espirometría en más de 9.000 adultos de 14 países, se encontró que el 96 % de las pruebas cumplía con los objetivos de calidad y se sugirió que la capacitación previa de los técnicos influía de forma significativa en dichos resultados. Este hallazgo es similar a lo observado en el presente estudio al utilizar la estrategia de capacitación para perfeccionar la ejecución de las pruebas.

En los estudios de Araya *et al.*^16^ y de van de Hei *et al.*^17^ se observó que el 77,1 % de las pruebas presentaba más de un error en los criterios de aceptabilidad, como esfuerzo variable, terminación temprana y pico de flujo espiratorio mayor del 10 %. En menor proporción, se observó incumplimiento de los criterios de repetibilidad, lo cual es comparable con los resultados de la presente investigación.

Como conclusión se destaca que, a medida que avanzaron las sesiones, se optimizó la calidad de las espirometrías forzadas debido al perfeccionamiento de la técnica para practicar las pruebas y a la capacitación continua de los profesionales encargados.

La principal limitación del estudio se relaciona con la variabilidad en la estandarización de la técnica y la observancia de los estándares internacionales para practicar la prueba con el fin de garantizar resultados válidos, reproducibles y comparables. Esto implica cumplir con el acondicionamiento y la calibración de los equipos, ejecutar adecuadamente la maniobra, y seguir los criterios de calidad, aceptabilidad y repetibilidad de la ATS/ERS.
